# A broad *v.* focused digital intervention for recurrent binge eating: a randomized controlled non-inferiority trial

**DOI:** 10.1017/S0033291722001477

**Published:** 2023-07

**Authors:** Jake Linardon, Adrian Shatte, Zoe McClure, Matthew Fuller-Tyszkiewicz

**Affiliations:** 1 School of Psychology, Deakin University, 1 Gheringhap Street, Geelong, VIC 3220, Australia; 2 Center for Social and Early Emotional Development, Deakin University, Burwood, Victoria, 3125, Australia; 3 Federation University, School of Engineering, Information Technology & Physical Sciences, Melbourne, Australia

**Keywords:** Binge eating, bulimia nervosa, digital intervention, eating disorders, e-health, randomized trial, smartphone app

## Abstract

**Background:**

Empirically validated digital interventions for recurrent binge eating typically target numerous hypothesized change mechanisms via the delivery of different modules, skills, and techniques. Emerging evidence suggests that interventions designed to target and isolate one key change mechanism may also produce meaningful change in core symptoms. Although both ‘broad’ and ‘focused’ digital programs have demonstrated efficacy, no study has performed a direct, head-to-head comparison of the two approaches. We addressed this through a randomized non-inferiority trial.

**Method:**

Participants with recurrent binge eating were randomly assigned to a broad (*n* = 199) or focused digital intervention (*n* = 199), or a waitlist (*n* = 202). The broad program targeted dietary restraint, mood intolerance, and body image disturbances, while the focused program exclusively targeted dietary restraint. Primary outcomes were eating disorder psychopathology and binge eating frequency.

**Results:**

In intention-to-treat analyses, both intervention groups reported greater improvements in primary and secondary outcomes than the waitlist, which were sustained at an 8-week follow-up. The focused intervention was not inferior to the broad intervention on all but one outcome, but was associated with higher rates of attrition and non-compliance.

**Conclusion:**

Focused digital interventions that are designed to target one key change mechanism may produce comparable symptom improvements to broader digital interventions, but appear to be associated with lower engagement.

## Introduction

Binge eating is a symptom common across many subthreshold and diagnostic-level eating disorders. Although evidence-based treatment and prevention programs for binge eating exist (Hilbert et al., 2019), there remains a significant gap in the uptake of these services among those in need (Weissman & Rosselli, 2017). The reasons for this service gap include the high cost of mental health services, limited professional availability and lengthy waitlists, geographical constraints, and percieved stigma (Kazdin, Fitzsimmons-Craft, & Wilfley, 2017). If unaddressed, the presence of binge eating can lead to a clinically significant eating disorder or numerous adverse complications (Klump, Bulik, Kaye, Treasure, & Tyson, 2009). Thus, solutions that reduce this service gap are sorely needed.

One possible solution is to deliver intervention content through technological mediums, such as the Internet or smartphone apps. Digital interventions are advantageous because they can reach a large number of people at little to no cost, and can be completed at home, anonymously, and at a self-suited pace (Andersson, 2016). While many digital programs require professional guidance, the utility of self-guided digital interventions is becoming more widely recognized. Self-guided digital interventions are not only more disseminable, but technological advancements means that some features that characterize the client-therapist relationship (tailored content delivery, assessment of risk profile etc.) can be mirrored through in-built app functionality, such as conversational agents, anonymous online screening, and just-in-time intervention prompts (Fitzsimmons-Craft et al., 2021; Torous et al., 2021). Despite producing smaller effects than professionally guided programs (Baumeister, Reichler, Munzinger, & Lin, 2014), the demand for self-guided digital interventions is growing among people with eating disorders (Linardon, Messer, Lee, & Rosato, 2021c). While self-guided programs are not the sole solution to the existing service gap, they can broaden the dissemination of evidence-based treatments and help more people than would have otherwise been the case in the absence of any intervention (Torous et al., 2021).

Existing digital programs for eating disorders typically involve numerous strategies, techniques, or modules designed to target a range of hypothesized change mechanisms, such as restrictive eating, mood dysregulation, body image concerns, and self-esteem deficits, (de Zwaan et al., 2017; Fitzsimmons-Craft et al., 2020). While these broad, ‘multi-target’ programs are effective for many, they are also limited in certain ways. Some users may not require a program that targets multiple mechanisms because they do not exhibit some of the problems that are being addressed (e.g. a person that does not experience body image concerns does not need intervention content or strategies designed to alleviate body concerns). Receiving intervention content that is not relevant to a user's symptom profile may lead to issues with motivation, engagement and drop-out (Andersson, Estling, Jakobsson, Cuijpers, & Carlbring, 2011).

Recent attention has been devoted toward developing more *focused* digital intervention formats. One example of this is the ‘single session’ intervention, which is an online program that incorporates one component of evidence-based treatment, targets one or two key change mechanisms, and requires only one encounter that program (Schleider, Dobias, Sung, Mumper, & Mullarkey, 2020). Single-session interventions are hypothesized to improve the acceptability and accessibility of digital health tools because, unlike multi-session formats, they can minimize engagement burdens on users (as they can be completed in only one sitting). Furthermore, many single session programs are cost-free and publicly accessible, which likely yields far greater reach and public health impact (Schleider et al., 2020). Importantly, single session online mental health interventions can produce effect sizes slightly smaller to multi-session interventions (Schleider & Weisz, 2017b).

Another example of a focused digital intervention format is a single-target program. Like a single-session intervention, single-target interventions are theoretically precise, mechanism-focused programs that addresses only one specific problem hypothesized to underlie an outcome (Linardon et al., 2021b). Such single-target, focused interventions are not typically completed in one sitting because they are multi-step programs that deliver more content and teach a broader range of skills. Even though such focused interventions take longer to complete than single-session interventions, compared to broad programs their degree of specificity may be more relevant to certain users. Further, if a focused intervention targets a mechanism known to underlie most of the effects of treatment, they might be just as beneficial as a broader program that targets numerous hypothesized mechanisms.

Evidence supports the efficacy of focused digital interventions for eating disorder symptoms. Multi-step, self-guided digital interventions designed to exclusively target maladaptive perfectionism (Shu et al., 2019) and dietary restraint (Linardon et al., 2021b) have been produced effect sizes comparable to broad programs. However, no study has directly compared a broad and focused program to determine their relative efficacy, as large adequately powered trials are difficult to execute. Establishing their relative efficacy through a non-inferiority trial would have significant implications for the future design, delivery, and dissemination of digital interventions for eating disorders.

We conducted a randomized non-inferiority trial comparing a broad to a focused digital intervention for recurrent binge eating. The broad program was designed to target three key binge eating maintaining mechanisms (dietary restraint, mood intolerance, and body image), while the focused program was designed to target one key change mechanism (dietary restraint). Both interventions have demonstrated efficacy (Linardon, Shatte, Rosato, & Fuller-Tyszkiewicz, 2020b; Linardon et al., 2021b), but their comparative efficacy has yet to be tested. A decision was made to isolate dietary restraint in the focused program as prior multisite trials have shown that the effects of traditional CBT for bulimia nervosa are most strongly mediated by early reductions in this mechanism as opposed to the other hypothesized mechanisms (Sivyer et al., 2020; Wilson, Fairburn, Agras, Walsh, & Kraemer, 2002). Thus, there is reason to suspect that a digital intervention exclusively designed to target dietary restraint may be non-inferior to a digital intervention designed to target multiple theorized change mechanisms.

It was hypothesized that participants randomized to either of the two digital interventions would experience greater improvements in primary and secondary outcomes than participants randomized to the waitlist. It was also hypothesized that the focused digital intervention would not be inferior to the broad digital intervention at the post-test and follow-up periods.

## Method

### Design

This study is a remote trial comparing three groups: a broad digital intervention, a focused digital intervention, and a waiting list. Assessments were conducted at baseline, 4-weeks post-randomization, and 8-weeks post-randomization. This trial received ethical clearance from Deakin University and was pre-registered (ACTRN12621000914864). All participants provided informed consent.

### Study population and recruitment

Participants were recruited in July-August 2021 via advertisements distributed throughout the first author's psychoeducational platform for eating disorders. This platform consists of an open-access website (https://breakbingeeating.com/) and social media accounts. It displays passive educational content related to eating disorders, including their causes, consequences, epidemiology, and help options. This platform contains passive information about eating disorders, rather than active, multi-step self-help programs. The majority of visitors do not have access to traditional forms of care and have reported using the platform to get some form of self-help information (Linardon, Rosato, & Messer, 2020a), rendering this a suitable target population.

Respondents to advertisements first completed a screening survey to determine their eligibility. Participants were eligible if they (1) were aged 18 years or over, (2) had access to the Internet and a smartphone, and (3) self-reported the presence of recurrent objective binge eating, defined as one episode per every two weeks, on average, over the past three months. Participants who met eligibility criteria then completed baseline assessments.

### Randomization

**Fig. 1.**
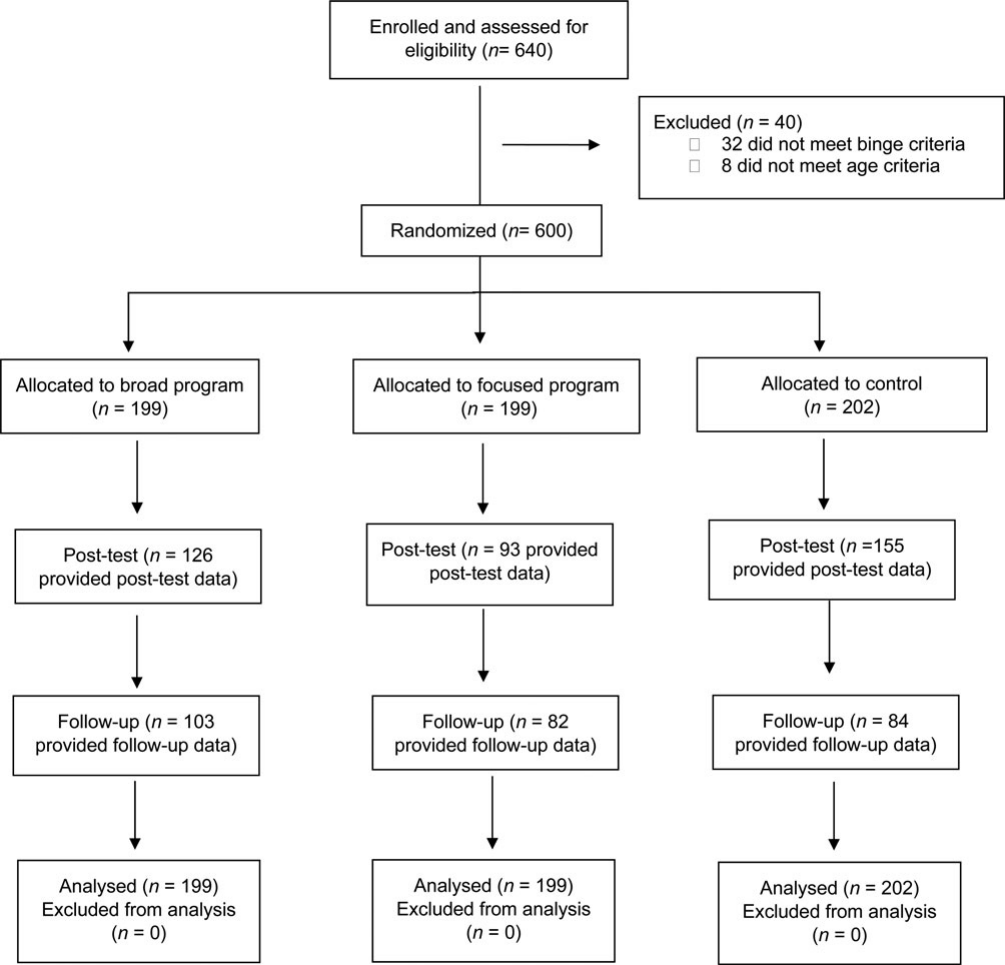
Flow of participants throughout the study.

Participants were randomized into one of three groups in a 1:1:1 ratio generated through an automated computer-based random number sequence provided in Qualtrics. Upcoming allocations were concealed from the researchers and participants as the randomization process was entirely automated. Six-hundred participants were randomized (see Fig. 1).

### Study conditions

We implemented a user-centered design framework when developing the digital interventions. End-users were involved in the conception, design, and testing of the interventions through a series of phases. In Phase 1, the target population was surveyed to understand their receptiveness to and attitudes toward digital interventions, preferred functionality, and content delivery formats (Linardon, Shatte, Tepper, & Fuller-Tyszkiewicz, 2020c; Linardon et al., 2021c). In Phase 2, digital intervention content, functionality, and layout were developed, with its usability evaluated in a small sample of end-users (Linardon, King, Shatte, & Fuller-Tyszkiewicz, 2021a). In Phase 3, the acceptability and preliminary efficacy of the two digital interventions were tested (Linardon et al., 2021b).

#### Broad intervention

The broad program, *Break Binge Eating*, sought to address three hypothesized binge eating maintaining mechanisms: dietary restraint, mood dysregulation, and body image concerns. Intervention content was based on Fairburn's (2008) transdiagnostic CBT protocol. There were four modules in total, the first being psychoeducational and the remaining three dedicated toward targeting one maintaining mechanism (see Table 1 for a full description). Although participants were encouraged to stay on one module and practice its exercises for one week before moving on, the self-guided nature of this intervention meant that the participant could decide on the speed of their progression.

**Table 1.** Description of the intervention programs

Program	Module/Session	Topics covered	Recommended duration	Homework exercises & other features
**Broad program**				
	Psychoeducation	Definition of binge eating and how it differs to overeating. Different types of binge episodes (subjective *v.* objective) Factors that account for the persistence of binge eating: Dietary restraint Mood dysregulation Body image disturbances	1 day	Multiple choice quiz Word association test EMA symptom tracking and progress monitoring features (completed daily)
	Targeting dietary restraint	Why dietary restraint can maintain binge eating How real-time monitoring of eating habits can help the person gain insight into the role of restrictive eating on binge eating What is needed to be recorded in this context What a completed self-monitoring form looks like The importance of establishing regular eating and how it combats restrictive and delayed eating Key regular eating guidelines How to avoid grazing behavior	Approx. 7 days	Digital food diary Separate daily diary to plan next day's regular eating schedule Catalogue of activity ideas to engage in in attempt to avoid grazing behavior.
	Targeting mood dysregulation	Why mood dysregulation can maintain binge eating How and why acceptance and mindfulness skills can combat emotionally-charged binge eating episodes Systematic problem-solving as a tool to prevent the onset of those adverse experiences that trigger binge eating.	Approx. 7 days	Guided meditation recordings Deep breathing recording exercises Quick mood boosters (i.e. visualizing happiness, de-stress with a body scan) Practicing the six steps to effective problem solving using Jane's example
	Targeting body image concerns	Why body image problems can indirectly maintain binge eating Placing too much importance on your body image Why broadening your scheme of self-worth is important Societal ideals of the perfect body image Focusing on the positives Appreciating the functionality of the body	Approx. 7 days	Three-step exercise to record a list of activities to pursue that are independent of body image (e.g. gaming). Adding a rebuttal to made-up scenarios that reinforce appearance ideals. Functionality appreciation exercise
**Focused program**				
	Psychoeducation	Binge eating: what it is and the different episodes experienced. How dietary restraint can maintain binge eating Characteristics of harmful weight loss diets Mechanisms linking restrictive eating to binge eating Different types of food rules What a life without dieting can look like	1 day	Multiple choice quiz (hosted via the web platform). EMA symptom tracking and progress monitoring features (completed daily in the app component)
	Implementing self-monitoring	How real-time monitoring of eating habits can help you gain insight into the role of restrictive eating on binge eating What is needed to be recorded in this context What a completed self-monitoring form looks like	Approx. 7 days	End of session quiz (web platform). Digital food diary (app feature)
	Regular eating	The importance of establishing regular eating and how it combats restrictive and delayed eating Key regular eating guidelines How to avoid grazing behavior	Approx. 7 days	Separate daily diary to plan next day's regular eating schedule (app feature) Guided breathing exercise to surf the urge to binge (app feature)
	Exposure to feared foods	Why avoiding foods can contribute to binge eating Graded exposure as a tool to eliminate food anxiety and test underlying beliefs How to implement a brief, exposure-based food exercise.	Approx. 7 days	Three step exposure exercise (app feature) Create hierarchy of feared foods Select food to incorporate back into one's regime Test the validity of the belief and reflect on it after a few days

*Break Binge Eating* was delivered through a smartphone app. Its content was presented via audio recordings, written text, and graphics. It took users between 30 and 60 min to go through each module, depending on how quickly the material was learnt. Alongside the main content included interactive in-built app features, such as quizzes, a digital self-monitoring diary, symptom tracking, and text boxes to complete required homework activities. One noteworthy feature was the progress monitoring feature. This feature involved an end-of-day prompt asking participants to record the number of binge eating episodes experienced. If a participant responded to this prompt, the app would graph the user's daily binge episodes into a bar-chart so that their progress could be visualized over the last 10 days. This symptom tracking feature was included to maintain accountability and potentially enhance motivation.

#### Focused intervention

The focused program, *Breaking the Diet Cycle,* sought to address one hypothesized maintaining mechanism: dietary restraint. This program was also based on established CBT protocols (Fairburn, 2008). Content was divided into four sessions. Each session taught the participant one key strategy designed to modify dietary restraint. Session one was psychoeducational in nature, while sessions two, three, and four respectively taught users skills related to real-time self-monitoring, adopting regular eating, and overcoming food anxiety. Participants were also provided guidance on how long they should remain on one session before moving onto the next session (see Table 1). However, participants had the option of going at a self-suited pace.

*Breaking the Diet Cycle* was delivered through both a web portal and smartphone app. The web portal hosted session content, including written text, video tutorials, and graphics explaining the skills to be learnt, why they are important, and their successful implementation. In the pre-registered protocol, we stated that each session would take 30–60 min; however, participants likely completed each session in a shorter time frame given the amount of content provided. In each web session, participants were encouraged to practice the prescribed strategies via several homework exercises. These homework exercises were presented in the app component of the intervention, which allowed users to practice these skills digitally and in their daily life. For example, the app contained a digital food dairy, allowing participants to monitor their eating behaviors in real-time (as taught in session two). Importantly, the app did not contain additional content; it only helped participants practice the skills taught in the web sessions.

In both groups, participants were sent reminder emails every two weeks encouraging continued program use, and guidance was provided on how long it should take for participants to progress through the program. Participants were not reimbursed.

#### Control group

Control participants were placed on a waitlist and completed the same study assessments. After completing the post-test survey, control participants were given access to intervention content.

### Study assessments

#### Participant characteristics

At baseline, participants indicated their age, gender, ethnicity, education level, and current treatment status. Participants also self-report whether they had a current or prior eating disorder or other mental health disorder, as diagnosed by a professional (yes *v.* no response). Motivation to change was assessed via asking participants to rate the extent to which they are motivated to change their disordered eating habits. Confidence was also assessed via asking participants to rate the extent to which they are confident in their ability to change their disordered eating habits. Both items were assessed via a visual analog scale, ranging from 1 (*not at all motivated/confident*) to 10 (*extremely motivated/confident*).

#### Primary outcomes

The two pre-registered primary outcomes were the global score (Cronbach's *α* = 0.88) from the Eating Disorder Examination Questionnaire (Fairburn & Beglin, 1994) and the frequency of objective binge eating. The global score is calculated by averaging the four EDE-Q subscales, which includes 22 items rated along a 7-point scale. Objective binge eating frequency was assessed via asking participants to indicate the number of episodes experienced over the past 28 days.

#### Secondary outcomes

Secondary outcomes included the shape concern (*α* = 0.83), weight concern (*α* = 0.73), eating concern (*α* = 0.72), and dietary restraint (*α* = 0.80) subscales from EDE-Q, and items assessing the frequency of subjective binge eating and compensatory behaviors experienced over the past 28 days. Compensatory behavior frequency was operationalized as the average number of self-induced vomiting, laxative use, and driven exercise episodes experienced over the past month. General psychological distress was also assessed via the total score (*α* = 0.86) from the Patient Health Questionnaire-4 (Kroenke, Spitzer, Williams, & Löwe, 2009).

### Sample size calculation

Sample size was calculated based on non-inferiority tests, as these require larger samples than for standard superiority testing. Based on a recent efficacy trial with the broad digital program used in this study (Linardon et al., 2020b), the efficacy for primary outcomes was expected to be *d* > 0.5, for which a non-inferiority limit of *d* = 0.25 was derived for powering the non-inferiority evaluation. This limit of *d* = 0.25 constitutes a preserved fraction of 50%, which is common in non-inferiority trials (Althunian, de Boer, Groenwold, & Klungel, 2017), and also represents a small but meaningful group difference that may be expected to be of clinical significance. Setting power at 0.80 and alpha at 0.05 (one-tailed), the required sample size per intervention arm was 198. Thus, our target sample size at baseline was 198 per group, which also ensured adequate power to test for differences between the control group and each of the intervention groups for whom effect sizes were expected to be larger than the non-inferiority limit.

### Statistical analyses

Analyses were undertaken using Stata version 16, and followed intention-to-treat principles by retaining participants in the condition they were randomized to at baseline. In these models, missing data were handled using multiple imputations with 50 imputations derived via the fully conditional specification method. Results of subsequent analyses on each imputed dataset were pooled using Rubin's (1987) rules. We also conducted sensitivity analyses using the last observation carried forward method. Findings pertaining to these sensitivity analyses are presented in online Supplementary Materials.

Linear mixed models were used for hypothesis testing of outcome measures, except binge eating and compensatory behavior frequency where Poisson mixed models were used. All models included repeated measures (baseline to post-test) clustered within individuals. Comparison between the two intervention arms and control group participants were limited to baseline *v.* post-test time-points as control participants were given access to the intervention after post-test. Evaluations of change from post-intervention to follow-up were conducted for, and compared between, the two intervention groups

For continuous outcomes, effect sizes are reported as standardized mean differences, with values of 0.20 considered small, 0.50 moderate, and 0.80 and above considered large (Cohen, 1992). For count outcomes, risk ratios (RR) were instead used. RR values of 1 indicate no difference in change in outcome count scores across groups (baseline to post-test comparisons) or time (post-test to follow-up). RR values <1 indicate reduction in binge eating and compensatory behavior outcomes over time (post-test *v.* follow-up) or for either of the intervention groups relative to control condition (post-test differences). RR <0.60 may be considered small, RR <0.29 moderate, and RR <0.15 large (Chen, Cohen, & Chen, 2010).

## Results

### Baseline characteristics

Table 2 presents the characteristics of participants at baseline. Most participants were White, educated, women. The three groups did not differ on any baseline variable, indicating that randomization was successful.

**Table 2.** Baseline characteristics of all randomized participants

Variable	Control group (*n* = 202)	Broad intervention (*n* = 199)	Focused intervention (*n* = 199)	Test statistic	*ES*
Age	34.11 (10.24)	34.02 (9.45)	33.41 (9.79)	0.30	0.00
Gender (female)	192 (95.0%)	185 (93.0%)	186 (93.5%)	0.82	0.03
Ethnicity				5.10	0.06
Caucasian	181 (89.6%)	172 (86.4%)	170 (85.4%)		
Multiracial	3 (1.5%)	4 (2.0%)	4 (2.0%)		
Asian	6 (3.0%)	8 (4.0%)	9 (4.5%)		
Black	4 (2.0%)	3 (1.5%)	8 (4.0%)		
Other	8 (4.0%)	12 (6.0%)	8 (4.0%)		
Education level				7.10	0.07
Did not finish secondary school	1 (0.5%)	4 (2.0%)	0 (0%)		
Year 12/senior year or equivalent	20 (9.9%)	14 (7.0%)	13 (6.5%)		
Higher than year 12/senior	181 (89.6%)	181 (91.0%)	186 (93.5%)		
Past AN	23 (11.4%)	14 (7.0%)	14 (7.0%)	3.26	0.07
Past BN	22 (10.9%)	27 (13.6%)	27 (13.6%)	0.86	0.03
Past BED	57 (28.2%)	60 (30.2%)	52 (26.1%)	0.79	0.03
Past OSFED	8 (4.0%)	7 (3.5%)	10 (5.0%)	0.59	0.03
Current eating disorder				5.56	0.06
AN	3 (1.5%)	3 (1.5%)	0 (0%)		
BN	10 (5.0%)	14 (7.0%)	13 (6.5%)		
BED	53 (26.2%)	48 (24.1%)	42 (21.1%)		
OSFED	7 (3.5%)	7 (3.5%)	9 (4.5%)		
Past MDD	56 (27.7%)	67 (33.7%)	46 (23.1%)	5.50	0.09
Past anxiety disorder	79 (39.1%)	96 (48.2%)	79 (39.7%)	4.27	0.08
Past SUD	6 (3.0%)	8 (4.0%)	9 (4.5%)	0.68	0.03
Current MDD	35 (17.3%)	37 (18.6%)	25 (12.6%)	2.97	0.07
Current anxiety disorder	58 (28.7%)	66 (33.2%)	56 (28.1%)	1.43	0.04
Current SUD	1 (0.5%)	3 (1.5%)	1 (0.5%)	1.63	0.05
Receiving current treatment	43 (21.3%)	42 (21.1%)	35 (17.6%)	1.08	0.04
Motivation level	8.80 (1.41)	8.84 (1.43)	8.79 (1.45)	0.08	0.00
Confidence level	5.72 (2.22)	5.48 (2.18)	5.60 (2.27)	0.58	0.00
OBE frequency	16.73 (15.73)	16.27 (12.98)	17.30 (14.17)	0.25	0.00
EDE-Q global	3.92 (0.97)	3.93 (1.03)	4.02 (0.97)	0.71	0.00
EDE-Q restraint	3.05 (1.68)	3.05 (1.50)	3.15 (1.52)	0.29	0.00
EDE-Q shape concern	4.76 (1.04)	4.74 (1.23)	4.88 (1.05)	0.93	0.00
EDE-Q weight concern	4.27 (1.05)	4.31 (1.16)	4.39 (1.14)	0.58	0.00
EDE-Q eating concern	3.59 (1.18)	3.62 (1.26)	3.68 (1.24)	0.27	0.00
SBE frequency	15.02 (17.73)	14.71 (17.30)	17.43 (20.93)	1.26	0.00
Compensatory behaviors	3.84 (7.57)	5.25 (8.44)	4.86 (7.41)	1.74	0.01
PHQ-4 psychological distress	6.24 (3.38)	6.22 (3.16)	6.49 (3.16)	0.43	0.00

AN, anorexia nervosa; BN, bulimia nervosa; BED, binge-eating disorder; OSFED, other specified feeding or eating disorder; MDD, major depressive disorder; SUD, substance use disorder; EDE-Q, Eating Disorder Examination Questionnaire; = Patient Health Questionnaire; Test statistic, F-statistic from ANOVA for continuous variables and *χ*^2^ tests for categorical variables; ES, effect size. Effect size presented as Cohens *d* for continuous variables and phi coefficient for categorical variables.

* *p* < 0.05.

### Study attrition

A total of 374 participants provided data on one of the two primary outcomes at post-test and 269 provided data on one of the two primary outcomes at follow-up. Three-hundred-fifty-nine participants provided primary outcome data on at least one assessment. The three groups differed on post-test attrition rates (*χ*^2^ = 38.54. *p* < 0.001, *ϕ* = 0.25), with the control group (*n* = 47; 23%) associated with lower attrition at post-test than the broad (*n* = 73; 36%) and focused group (*n* = 106; 53%). The broad intervention group was associated with a lower attrition rate at post-test than the focused group (*p* = 0.001). There was no group difference (*p* = 0.056) on attrition rates at the follow-up period between the three conditions (58% for control, 48% for broad, and 58% for focused group). Drop-outs were younger (*d* = 0.19, *p* = 0.019), and reported more frequent subjective binge episodes (*d* = 0.17, *p* = 0.049) and compensatory behaviors (*d* = 0.19, *p* = 0.024).

### Intervention usage

#### Broad intervention

The uptake rate (defined as at least one login) for the broad intervention group was high, with 171 participants (85.9%) logging in at least once. Of those who accessed the intervention, 86% completed at least 50% of the content from Module 1, 66% for Module 2, 48% for Module 3, and 31% for Module 4. 59% completed at least 50% of the content within the program. The mean number of modules completed was 2.32 (s.d. = 1.43), the mean number of self-monitoring diary entries was 24.23 (s.d. = 43.97), and the mean number of days the app was used was 13.14 (s.d. = 9.95).

#### Focused intervention

One-hundred-sixty-four (82%) participants downloaded the focused program. Of those who accessed the intervention, 48% of participants completed at least 50% of program content, with a mean of 1.95 sessions (s.d. = 1.62) completed. Of those who accessed the app component (*n* = 134), the mean number of self-monitoring diary entries was 16.03 (s.d. = 36.09), and the mean number of days the app was used was 7.42 (s.d. = 7.75).

*Group Comparisons*. The two groups did not differ on uptake rates (*p* = 0.336). However, when including all randomized participants (i.e. even those who did not log in to their program), compared to the focused group, the broad group was associated with higher rates of adherence (⩾50 content completed; 50% *v.* 39%, *p* = 0.027, *ϕ* = 0.11) and greater number of modules/sessions completed (*p* = 0.018, *d* = 0.23).

### Post-test efficacy

#### Primary outcomes

Results from the intention-to-treat analyses comparing the three groups on primary outcomes are presented in Table 3. When comparing the control group with the two intervention groups, the mean differences in objective binge eating frequency and EDE-Q global scores were statistically significant. In both cases, the intervention groups reported greater reductions in primary outcomes than the control group. However, there were no differences in the degree of change on primary outcomes between the two intervention groups, with criteria for non-inferiority (difference in *d* < 0.25) being satisfied. Online Supplementary Fig. S1 presents a graphical representation of rate of change in primary outcomes across the study conditions.

**Table 3.** Means, Standard Deviations, and change scores on primary and secondary outcomes across the three conditions

Outcome	Baseline		Post-test			Change score difference			
	*M* (s.d.)	*n*	*M* (s.d.)	*n*	*ES*_within_	Comparison	*M* change (95% CI)	*ES*_between_	*p*
EDE-Q global									
Control	3.92 (0.97)	202	3.85 (0.96)	155		Broad *v.* control	−0.74 (−0.96 to −0.53)	−0.74	<0.001
Broad intervention	3.93 (1.03)	199	3.06 (1.14)	122	−1.29*	Focused *v.* control	−0.89 (−1.14 to −0.65)	−0.89	<0.001
Focused intervention	4.03 (0.98)	199	2.93 (1.27)	92	−1.33*	Broad *v.* Focused	−0.15 (−0.43 to 0.12)	−0.15	0.280
OBE frequency									
Control	16.73 (15.74)	202	17.78 (16.16)	155		Broad *v.* control	−0.50 (−0.68 to −0.31)	0.61	<0.001
Broad intervention	16.28 (12.99)	199	10.30 (6.95)	126	0.64*	Focused *v.* control	−0.52 (−0.74 to −0.31)	0.59	<0.001
Focused intervention	17.31 (14.18)	199	10.62 (10.69)	95	0.58*	Broad *v.* Focused	−0.03 (−0.25 to 0.19)	0.97	0.782
SBE frequency									
Control	15.02 (17.74)	202	16.59 (19.08)	155		Broad *v.* control	−0.36 (−0.69 to −0.02)	0.70	0.037
Broad intervention	14.71 (17.30)	199	10.07 (8.56)	126	0.95	Focused *v.* control	−0.59 (−0.96 to −0.20)	0.56	0.002
Focused intervention	17.44 (20.94)	199	9.57 (10.69)	95	0.70*	Broad *v.* Focused	−0.23 (−0.60 to 0.14)	0.79	0.220
Compensatory behaviors									
Control	3.84 (7.57)	202	4.22 (9.09)	155		Broad *v.* control	−1.24 (−1.57 to −0.90)	0.29	<0.001
Broad intervention	5.26 (8.45)	199	1.35 (2.83)	122	0.50*	Focused *v.* control	−0.77 (−1.10 to −0.43)	0.46	<0.001
Focused intervention	4.87 (7.42)	199	2.01 (3.58)	92	0.64*	Broad *v.* Focused	0.47 (0.12–0.81)	1.59	0.009
EDE-Q shape concerns									
Control	4.76 (1.05)	202	4.68 (1.01)	155		Broad *v.* control	−0.66 (−0.92 to −0.40)	−0.58	<0.001
Broad intervention	4.75 (1.23)	199	3.92 (1.38)	122	−1.01*	Focused *v.* control	−0.88 (−1.17 to −0.59)	−0.77	<0.001
Focused intervention	4.89 (1.06)	199	3.76 (1.61)	92	−1.12*	Broad *v.* Focused	−0.22 (−0.55 to 0.11)	−0.19	0.189
EDE-Q weight concerns									
Control	4.28 (1.06)	202	4.23 (1.03)	155		Broad *v.* control	−0.56 (−0.81 to −0.30)	−0.50	<0.001
Broad intervention	4.31 (1.17)	199	3.67 (1.25)	122	−0.84*	Focused *v.* control	−0.68 (−0.97 to −0.39)	−0.61	<0.001
Focused intervention	4.39 (1.14)	199	3.55 (1.58)	92	−0.82*	Broad *v.* Focused	−0.12 (−0.45 to 0.20)	−0.10	0.451
EDE-Q eating concerns									
Control	3.59 (1.19)	202	3.42 (1.25)	155		Broad *v.* control	−0.84 (−1.12 to −0.55)	−0.68	<0.001
Broad intervention	3.63 (1.27)	199	2.57 (1.44)	122	−1.18*	Focused *v.* control	−1.01 (−1.31 to −0.70)	−0.82	<0.001
Focused intervention	3.68 (1.25)	199	2.43 (1.42)	92	−1.34*	Broad *v.* Focused	−0.17 (−0.52 to 0.17)	−0.13	0.325
EDE-Q dietary restraint									
Control	3.05 (1.68)	202	3.07 (1.54)	155		Broad *v.* control	−0.94 (−1.35 to −0.64)	−0.59	<0.001
Broad intervention	3.05 (1.50)	199	2.08 (1.49)	122	−1.09*	Focused *v.* control	−1.07 (−1.40 to −0.74)	−0.67	<0.001
Focused intervention	3.16 (1.53)	199	1.97 (1.45)	92	−1.03*	Broad *v.* Focused	−0.12 (−0.49 to 0.25)	−0.08	0.511
Psychological distress									
Control	6.24 (3.38)	202	6.22 (3.17)	155		Broad *v.* control	−0.82 (−1.46 to −0.19)	−0.25	0.011
Broad intervention	6.22 (3.17)	199	5.34 (3.14)	120	−0.36*	Focused *v.* control	−0.97 (−1.67 to −0.27)	−0.30	0.007
Focused intervention	6.49 (3.17)	199	5.68 (3.34)	92	−0.38*	Broad *v.* Focused	−0.15 (−0.91 to 0.61)	−0.05	0.702

M and s.d. values are based on non-imputed data; mean differences and effect sizes are derive from ITT analysis; ES, effect size; for objective and subjective binge, and compensatory behaviors the reported value is a risk ratio. For all other outcomes, effect size is a standardized mean difference.

*p < 0.05.

#### Secondary outcomes

When comparing the control group with the two intervention groups, the mean differences for each secondary outcome were significant (Table 3). In all cases, the intervention groups reported greater reductions in secondary outcomes than the control group. When comparing the two intervention groups, the only significant difference to emerge was on compensatory behavior frequency, with the broad intervention group reporting greater reductions in compensatory behaviors than the focused group. No other differences in secondary outcomes were observed between the two intervention groups.

### Follow-up

The degree of change between the two intervention groups from the post-test to follow-up period on primary and secondary outcomes is presented in Table 4. For all outcomes, initially achieved changes from baseline to post-test were sustained at follow-up for both intervention groups. However, compared to the broad group, the focused intervention group experienced significantly greater reductions from post-test to follow-up on compensatory behaviors and dietary restraint. No other between-group differences emerged at follow-up, with criteria for non-inferiority being satisfied.

**Table 4.** Comparison between app and web group at follow-up on primary and secondary outcomes

Outcome							Difference in post-test to follow-up change score			
	Broad intervention			Focused intervention			Focused – Broad			
	*n*	*M*	s.d.	*n*	*M*	s.d.	*M* change	95% CIs	*ES*_between_	*p*
EDE-Q Global score										
Post-intervention	122	3.06	1.14	92	2.93	1.27				
Follow-up	97	2.83	1.2	81	2.66	1.32	0.03	−0.33 to 0.27	−0.03	0.983
OBE frequency										
Post-intervention	126	10.3	6.95	95	10.62	10.69				
Follow-up	103	10.5	12.96	82	10.87	9.53	0.00	−0.30 to 0.30	1.00	0.983
SBE frequency										
Post-intervention	126	10.07	8.56	95	9.57	10.69				
Follow-up	103	10.00	14.30	82	9.52	10.02	0.17	−0.24 to 0.57	1.18	0.418
Compensatory behaviors										
Post-intervention	122	1.35	2.83	92	2.01	3.58				
Follow-up	97	1.14	2.44	81	1.52	2.79	−0.34	−0.64 to −0.03	0.71	0.029
EDE-Q Shape concerns										
Post-intervention	122	3.92	1.38	92	3.76	1.61				
Follow-up	97	3.59	1.63	81	3.48	1.68	0.08	−0.30 to 0.46	0.07	0.694
EDE-Q Weight concerns										
Post-intervention	122	3.67	1.25	92	3.55	1.58				
Follow-up	97	3.38	1.37	81	3.12	1.66	−0.08	−0.44 to 0.27	−0.07	0.649
EDE-Q Eating concerns										
Post-intervention	122	2.57	1.44	92	2.43	1.42				
Follow-up	97	2.13	1.38	81	2.28	1.46	0.26	−0.11 to 0.63	0.21	0.172
EDE-Q Restraint										
Post-intervention	122	2.08	1.49	92	1.97	1.45				
Follow-up	97	2.20	1.47	81	1.76	1.38	−0.40	−0.78 to −0.01	−0.26	0.042
Psychological distress										
Post-intervention	120	5.34	3.14	92	5.68	3.34				
Follow-up	95	5.18	3.24	80	4.76	3.07	−0.52	−1.42 to 0.39	−0.16	0.258

M and s.d. values are based on non-imputed data; mean differences and effect sizes are derive from ITT analysis; ES, effect size; for objective and subjective binge, and compensatory behaviors the reported value is a risk ratio. For all other outcomes, effect size is a standardized mean difference. OBE, objective binge eating; SBE, subjective binge eating.

## Discussion

We conducted a randomized non-inferiority trial comparing a broad and focused self-guided digital intervention for recurrent binge eating. Both interventions produced greater reductions in eating disorder symptoms than the control group. The magnitude of effects was unexpectedly comparable to recent trials of guided digital interventions (Fitzsimmons-Craft et al., 2020) and traditional psychological treatments (Hilbert et al., 2019) for eating disorders. This is likely explained by different lengths of follow-up assessment. Whereas recent trials of guided or therapist-led treatments conducted follow-up assessments as long as 8 months post-randomization (Fitzsimmons-Craft et al., 2020), our follow-up assessment occurred at a time where rapid, large reductions in core symptoms are often observed (Linardon, Brennan, & de la Piedad Garcia, 2016). Perhaps effects diminish as follow-up length increases.

We found evidence that the focused program was not inferior to the broad program on any symptom measure. It is noteworthy that no between-group differences were observed in those outcomes that were not a direct target of the focused intervention (but were in the broad intervention). Perhaps evidence of equivalence can be explained by the self-perpetuating nature of eating disorder symptoms. According to Fairburn's (2008) model of hypothesized feedback loops, extreme concerns with eating, weight and shape are both precipitants and consequences of restrictive and binge eating episodes, and engagement of disordered eating induces distress via the experience of shame and guilt. Thus, it is possible that targeting binge eating through one hypothesized mechanism may be sufficient to induce change on other symptoms implicated in this cycle. This cascade effect might also explain why we observed later improvements in compensatory behaviors in the focused program, even though these behaviors were not a direct target.

Intervention effects of attrition were also examined. While attrition was high for both intervention groups, the rates reported here are consistent with the attrition rate estimated in a recent meta-analysis of fully-remote, self-guided mental health app trials (Linardon & Fuller-Tyszkiewicz, 2020). A likely explanation for high attrition observed in fully remote trials is that participants who enroll via effortless online methods come to realize that remaining in the trial requires more effort than previously thought. In contrast, trials that require researcher consultation may attract more motivated participants and better allows the researcher to explain from the outset what is expected, potentially leading to greater retention. Furthermore, attrition was lower in the waitlist, which is also consistent with findings reported in existing meta-analyses (e.g. Linardon & Fuller-Tyszkiewicz, 2020) and individuals trials (Bakker, Kazantzis, Rickwood, & Rickard, 2018) of self-guided digital interventions. A possible interpretation of this is that, unlike those allocated to an immediate intervention group, those assigned to a waitlist are required to wait until after the follow-up assessment to gain access to program content, which could be a motivating factor to remain in the trial. Alternatively, perhaps those who did not engage with the interventions felt hesitant toward completing follow-up assessments asking about their experience of the program, resulting in the higher attrition found these groups.

The broad intervention group produced higher adherence and lower attrition than the focused group, suggesting that multi-step, focused programs like these may not yield the same engagement advantages observed in single session online interventions (Schleider & Weisz, 2017a). Trials of single-session interventions (which are also highly focused in nature) have produced rates of retention as high as 75% (Schleider et al., 2021), which is substantially greater than what was observed from our focused intervention. Perhaps the ability to complete the program in one sitting rather than focusing on one change mechanism is what affords single session interventions an engagement advantage over single-target interventions. Conversely, it is not fully understood why retention and adherence were higher for the broad group over the focused group. Perhaps the delivery of diverse program content accompanied by a large suite of different therapeutic techniques is better at enhancing user engagement. For example, someone allocated to a focused intervention might quickly disengage after not being receptive to the limited number of skills that are the key focus of the program, but this same person might persist with a broader program knowing that several other preferred techniques will be presented. Alternatively, it could be that the different device delivery formats between the two groups accounted for these effects. That is, accessing both a web and app platform may have presented an additional problem with usability for those allocated to the focused program, potentially explaining the lower engagement rates.

There are important limitations to this study. First, as the follow-up assessment was conducted 8-weeks post-randomization, the longer-term effects of these digital intervention formats are unknown. It is possible that the benefits observed from focused interventions diminish to a greater extent over longer follow-up periods. Examining the relative, long-term efficacy of focused and broad digital interventions is an important future direction.

Second, differential attrition and adherence rates between the two intervention groups may have in part been explained by the different digital delivery modes. Apps may hold distinct advantages over web programs because they (i) are always within arm's reach, (ii) enable users to perform and record exercises in their natural environment, and (iii) are thought to facilitate faster skill acquisition and utilization because they can be engaged with in different contexts (Bakker, Kazantzis, Rickwood, & Rickard, 2016). Although available trials directly comparing web and app programs have failed to identify key outcome differences (Stolz et al., 2018), we cannot rule out the possibility that observed differences found were in part attributable to different device delivery modes. Similarly, one of the exercises (forbidden food exposure) targeting dietary restraint was only presented in the focused program (all other exercises targeting restraint were the same between the two programs), potentially accounting for some of the observed effects. However, this exposure exercise was presented in the last session of the focused program (see Table 1), and considering that around 75% of participants dropped out prior to accessing this session, this difference between the two programs likely had a negligible impact on study findings.

Third, attrition was high. Although simulation studies indicate that multiple imputations provide unbiased parameter estimates even in the presence of large amounts of missing data (Madley-Dowd, Hughes, Tilling, & Heron, 2019), readers must take into account the amount of missing data when interpreting these findings. We note that re-running group difference tests under the assumption that people dropped out due to lack of symptom improvement led to predictable dampening of effect sizes, but all effects remained significant. Thus, we have some confidence in the robustness of the presented findings, but caution the true treatment effects may be somewhere between the conservative estimates in our re-analysis and those presented in-text.

Fourth, generalizability of findings is limited to White, well-educated, younger women. Attempts to recruit participants from other racial, gender, and socioeconomic groups are needed to better understand the role of digital interventions in different populations. Likewise, due to the self-reported nature of assessments, data on participant body mass were not collected. Body mass index may have moderated intervention effects, as has been shown previously (Vall & Wade, 2015), suggesting that consideration of this variable in future trials is necessary.

Present findings highlight the viability and clinical utility of both broad and focused formats of digital intervention for binge-spectrum eating disorders. Although digital interventions are not designed to replace traditional psychological treatment or completely resolve the existing service gap, we show that brief, low intensity, scalable online programs with different degrees of focus may be palatable options for many, including those who are either not interested in or cannot access traditional treatment approaches. We also show that focused programs designed to target one central change mechanism may be sufficient to induce meaningful change in other key eating disorder symptoms. A next step in research is to identify individual characteristics predictive of responsiveness to different digital intervention formats so that we can personalize the delivery of different intervention options for people with eating disorders.

## Supplementary material

For supplementary material accompanying this paper visit https://doi.org/10.1017/S0033291722001477. click here to view supplementary material
