# Supplementary material for: A broad v. focused digital intervention for recurrent binge eating: a randomized controlled non-inferiority trial
Source: Psychol Med. 2022 May 27;53(10):4580–91. doi: 10.1017/S0033291722001477 (PMC10388300; doi:10.1017/S0033291722001477)
Supplement: Supplementary file 1 [file S0033291722001477sup001.docx]

**Supplementary Material**

**Sensitivity analyses**

Sensitivity analyses were conducted for between group effects involving the wait-list control condition compared to the two intervention arms. As we did not follow up participants who dropped out to understand why they left the study, it is unclear whether their exit from the study was linked to lack of symptom improvement or other reasons unrelated to the intervention.

By carrying the last observation forward for individuals with missing data, we can test the effects on the results reported in Table 3 of the manuscript for potential bias due to participants dropping out of the study due to lack of symptom improvement. Below, we provide comparison data for the intervention arms vs control participants, providing statistics as presented in the manuscript and these revised analyses based on a strategy of last observation carried forward for missing data. We limit this re-analysis to comparisons involving the control group, for whom the intervention arms are expected to outperform. As a non-inferiority test framework was applied for comparisons among the intervention arms, and given the amount of data missing for both intervention arms, we felt that the last observation carried forward approach would skew conclusions in the direction of the non-inferiority hypothesis rather than offering a more meaningful ‘alternative’ estimate of between-group effects. Hence, we do not use this approach for comparisons among the two intervention arms.

As can be seen in Table A1 below, the mean change values and effect sizes decrease (as would be expected from using last value carried forward) in these re-analyses. Even so, all effects remained significant in these re-analyses, and hence would lead to the same substantive conclusion about treatment efficacy.

Table A1. *Comparison of original results based on multiple imputation against results using last observation carried forward.*

|  | Table 3 (multiple imputed) results | | | Last observation carried forward (LOCF) results | | |
| --- | --- | --- | --- | --- | --- | --- |
| Outcome | *M* change | ES | p | *M* change | ES | p |
| EDE-Q global |  |  |  |  |  |  |
| Broad vs control | -0.74 | -0.74 | <.001 | -0.41 | -0.41 | <.001 |
| Focused vs control | -0.89 | -0.89 | <.001 | -0.37 | -0.37 | <.001 |
| OBE frequency |  |  |  |  |  |  |
| Broad vs control | -0.50 | 0.61 | <.001 | -0.30 | 0.74 | <.001 |
| Focused vs control | -0.52 | 0.59 | <.001 | -0.22 | 0.78 | <.001 |
| SBE frequency |  |  |  |  |  |  |
| Broad vs control | -0.36 | 0.70 | .037 | -0.28 | 0.75 | .014 |
| Focused vs control | -0.59 | 0.56 | .002 | -0.34 | 0.71 | .003 |
| Compensatory behaviors |  |  |  |  |  |  |
| Broad vs control | -1.24 | 0.29 | <.001 | -0.60 | 0.55 | <.001 |
| Focused vs control | -0.77 | 0.46 | <.001 | -0.30 | 0.74 | .007 |
| EDE-Q shape concerns |  |  |  |  |  |  |
| Broad vs control | -0.66 | -0.58 | <.001 | -0.35 | -0.31 | <.001 |
| Focused vs control | -0.88 | -0.77 | <.001 | -0.35 | -0.31 | <.001 |
| EDE-Q weight concerns |  |  |  |  |  |  |
| Broad vs control | -0.56 | -0.50 | <.001 | -0.30 | -0.27 | .001 |
| Focused vs control | -0.68 | -0.61 | <.001 | -0.28 | -0.25 | .001 |
| EDE-Q eating concerns |  |  |  |  |  |  |
| Broad vs control | -0.84 | -0.68 | <.001 | -0.45 | -0.37 | <.001 |
| Focused vs control | -1.01 | -0.82 | <.001 | -0.40 | -0.33 | <.001 |
| EDE-Q dietary restraint |  |  |  |  |  |  |
| Broad vs control | -0.94 | -0.59 | <.001 | -0.55 | -0.35 | <.001 |
| Focused vs control | -1.07 | -0.67 | <.001 | -0.45 | -0.28 | <.001 |
| Psychological distress |  |  |  |  |  |  |
| Broad vs control | -0.82 | -0.25 | .011 | -0.46 | -0.14 | .035 |
| Focused vs control | -0.97 | -0.30 | .007 | -0.55 | -0.17 | .012 |


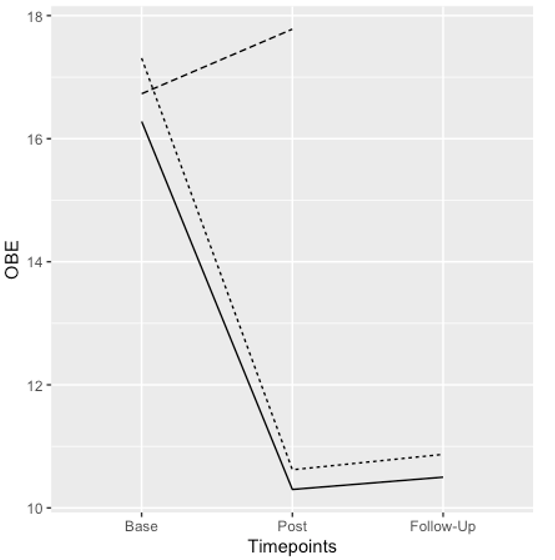

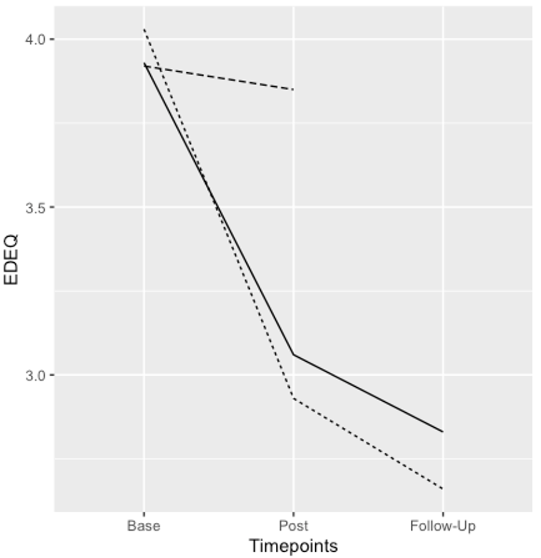


Supplementary Figure 1

Rate of change in the two primary outcomes (objective binge eating and EDE-Q global scores) across study conditions.

Thick line represents the broad digital intervention, thin shaded line represents the focused digital intervention and the thicker shaded line represents the control group (pre-posttest).
